# Navigating crisis: exploring the links between threat perceptions, well-being, individual and workplace resilience among general hospital staff

**DOI:** 10.1186/s13584-024-00656-2

**Published:** 2024-11-19

**Authors:** Chen Sharon Shmul, Baruch Berzon, Bruria Adini

**Affiliations:** 1 Emergency and Disaster Management Department, School of Public Health, Faculty of Medical and Health Sciences, Tel Aviv University, Tel Aviv, Israel; 2 Emergency Department and Emergency Preparedness, Assaf Harofeh Shamir Medical Center, Zriffin, Israel; 3 ResWell Research Collaboration, Tel Aviv University, Tel Aviv, Israel

**Keywords:** Psychological distress, Well-being, Individual resilience, Resilience at work, Perceived threats, Hospital

## Abstract

**Background:**

Hospital staff frequently encounter high-stress situations, emergencies, and disasters, which profoundly impact their well-being and resilience. The aim of the study was to examine associations between perceived threats, well-being, individual resilience, and resilience at work among staff of a general hospital, following the unexpected Hamas assault on Israel on October 7, 2023, and during the Israel-Gaza conflict.

**Methods:**

This cross-sectional study was conducted at a central Israeli public hospital, a level-two trauma center, surveying 434 staff members. Validated questionnaires were used to assess perceived threats, well-being, individual and work resilience, alongside demographic and professional characteristics. Data was collected via Qualtrics and paper questionnaires. Descriptive statistics, Pearson’s correlation, T-tests, ANOVA, Chi-square, and linear regression models were used to analyze relationships, differences, and key factors associated with well-being, personal resilience, and work resilience.

**Results:**

Key findings revealed that higher resilience at work and well-being are linked to greater individual resilience, while higher threat perception negatively affected well-being. Israeli-born individuals and those identifying as Jewish showed higher resilience. Men reported higher well-being than women, and physicians demonstrated higher well-being compared to nurses. Resilience at work was higher among administrative staff compared to nurses, with employment in the emergency department showing a significant negative relationship with resilience at work.

**Conclusions:**

The study revealed significant predictors of well-being, individual resilience, and workplace resilience among hospital staff in conflict situations. The immediate threat of war was perceived as most significant, highlighting the dynamic nature of threat perceptions. Prolonged emergencies can severely impact well-being, necessitating timely support. The findings emphasize the importance of integrated programs that enhance individual well-being and foster resilience in both personal and professional domains. Significant gender differences and the positive role of religiosity in resilience underscore the need for targeted interventions and systemic organizational changes to better support healthcare workers during crises. These insights highlight the importance of a comprehensive approach for cultivating a robust and resilient medical staff capable of effectively managing future crises.

## Introduction

Hospital staff are frequently exposed to stressful situations, emergencies, and life-threatening disasters [1]. The Centre for Research on the Epidemiology of Disasters (CRED) defines disasters as events that disrupt normal living conditions and cause a level of suffering that exceeds the affected community’s ability to cope, necessitating external (international) assistance [2]. Disasters are unexpected and often sudden events that result in significant damage, destruction, and human suffering [3]. They adversely affect the functioning of society, causing social, economic, and environmental damage. Some disasters are caused by natural events, while others are human-made, resulting from human actions, negligence, and errors [4].

Healthcare systems play a crucial role in responding to emergencies. When preparing for such situations, emphasis must be placed on the human factor and their well-being [5]. Well-being refers to individuals’ subjective assessment of their mental health, encompassing emotional, social, and psychological aspects. It serves as a measure of the individual’s overall quality of life [6]. A study conducted among 248 nurses in hospitals’ emergency departments in Belgium presented that these professionals are at increased risk of exposure to traumatic situations, leading to a decline in their well-being and overall functioning at work [7]. The COVID-19 pandemic significantly impacted health systems globally, particularly affecting the well-being of health workers on the front lines [8]. Well-being can be influenced by various factors, with individual resilience and threat perception being primary [9].

Individual resilience refers to an individual’s ability to adapt and cope with stress, distress, and challenges. It involves recovering from difficult experiences and maintaining a positive outlook in the face of unforeseen challenges. Individual resilience is essential for maintaining good mental health and well-being [10]. A literature review of 31 articles worldwide on the well-being of health workers during the COVID-19 crisis showed that those with higher individual resilience, effective coping strategies, and strong social support had better well-being [11]. A study examining the impact of a renewed outbreak of the COVID-19 pandemic on resilience and well-being found that while the pandemic significantly affected well-being, individual resilience played a protective role in mitigating adverse effects. Higher levels of resilience were associated with lower levels of distress and higher levels of well-being [12].

In contrast to individual resilience, resilience at work refers to an individual’s ability to cope with the demands, pressures, and challenges of the job or work environment [13]. This includes adapting to changes, recovering from setbacks, and maintaining productivity and performance in the face of unexpected challenges. Developing resilience at work can help employees maintain a positive outlook and improve their well-being [14]. Research indicates that exposure to emergencies is associated with lower levels of resilience at work, which is particularly important for health institution teams during high-demand and high-pressure emergencies [15]. A study conducted among 141 nurses in an Australian hospital found that personal characteristics impact nurses’ resilience and play a crucial role in determining resilience [16]. Individual and work resilience are not static traits but rather a set of skills and behaviors that can be developed and strengthened over time [17]. Psychosocial and cognitive interventions are essential for fostering resilience throughout life. Tailoring these interventions to the specific needs of the population can lead to improved well-being [18].

Threat perception also affects well-being. A study examining the relationship between the perceived threat of the Russia-Ukraine war and the well-being of medical staff found that health workers who perceived a higher threat were more likely to experience symptoms of depression and burnout. Differences in threat perception corresponded to the departments in which they were employed [19]. A study examining the relationship between the COVID-19 pandemic and the well-being and health of health workers in Pakistan found that those with a higher threat perception of the pandemic reported higher levels of anxiety and depression compared to those with a lower threat perception [20]. Another study conducted among 261 nurses in an emergency department in the Philippines examined the impact of threat perception from COVID-19 on well-being, job satisfaction, and intentions to leave their employment. The results showed that higher levels of fear were associated with decreased job satisfaction, increased psychological distress, and a higher likelihood of leaving the organization or profession [21]. Research conducted among approximately 333 nurses during the SARS outbreak focused on workplace threat perception and organizational support. It found a positive relationship between higher organizational support and lower threat perception [22].

Healthcare workers in Israel operate within an environment of constant exposure to threats, including ongoing conflicts, wars, and natural disasters [23]. In recent years, repeated hostilities, such as the Israel-Hamas conflict, involving rocket attacks, border skirmishes, and civil unrest, have directly affected the daily lives and mental health of citizens and frontline workers [24]. Additionally, Israel’s location along the seismically active Dead Sea Rift increases its susceptibility to earthquakes [25]. Moreover, cyber-attacks on healthcare facilities, like the breach at Hillel Yaffe Medical Center, have disrupted operations and endangered patient care. These multifaceted threats underscore the critical need for emergency preparedness, with an emphasis on ensuring the well-being and resilience of hospital staff to maintain effective functioning during future crises [26]. Hospitals must prepare for a variety of potential emergencies, including mass casualty events (MCIs), mass toxicological incidents (MTIs), unusual biological events (UBEs), cyber-attacks, earthquakes, and wars.

The aim of the study was to examine associations between perceived threats, well-being, individual resilience, and resilience at work among staff of a general hospital.

## Methods

### Study design

The current study was conducted in a public hospital in central Israel, which is a level-two trauma center. A representative sample of 434 staff members was surveyed from a total of 4,170 employees. The sample included staff from various hospital sectors such as medical, nursing including auxiliary staff, and administrative to ensure representation of the broader hospital workforce. Participants were recruited using a multi-step approach. First, Questionnaires were distributed electronically to all hospital employees across the specified sectors through institutional WhatsApp groups, with the approval of the hospital administration. Department heads were actively engaged to facilitate and encourage participation, ensuring that employees across different departments and shifts were reached. Data collection was managed using Qualtrics software. For staff members who were unable or unwilling to complete the questionnaire electronically, hard-copy versions were made available. These questionnaires were distributed and collected by designated coordinators within each department, who subsequently ensured the secure transfer of the completed questionnaires to the research team. All data from paper questionnaires were manually entered into the Qualtrics system, with double-entry verification procedures in place to ensure data accuracy and integrity. Participants who did not complete the questionnaire or whose responses were unclear regarding their group affiliation were excluded from the analysis. Ethical approvals for the study were granted by the Tel Aviv University Ethics Committee, approval number 0007363–2, dated November 5, 2023, and from the hospital’s Ethics Committee, approval number ASF-0251–23, dated November 8, 2023. Informed consent was obtained from all participants prior to their inclusion in the study.

### Study tools

The scales used in the current study were validated research tools previously employed in various studies. The questionnaire consisted of a number of measures that were combined together specifically for this study, as well as demographic and professional characteristics questions. All questionnaires were presented in their existing (validated) Hebrew versions.

#### Well-being questionnaire (World health organization (WHO-5)

In this study, the WHO-5 questionnaire was used to assess the individual’s well-being [27]. The WHO-5 consists of five items. Respondents were asked to rate the frequency with which they experienced each statement over the past two weeks on a 6-point Likert scale, from 0 (never) to 5 (all the time) [28]. The well-being score was calculated as the mean, with a Cronbach’s alpha of 0.83.

#### Individual resilience questionnaire (Davidson-Connor, 2003, CD-RISC 2)

Individual resilience was assessed using the short version of the Davidson-Connor Resilience Scale (CD-RISC 2) [29]. This scale has two items, with participants rating each item on a 5-point Likert scale, from 0 (not true) to 4 (almost always true) [30]. The final resilience score was the mean of the two items, and the Cronbach’s alpha was 0.72.

#### Resilience at work questionnaire (Hays, 2021, work resilience scale)

This study used the Work Resilience Scale developed by Hays in 2021 [31]. The questionnaire consists of 10 items that participants rate on a 5-point Likert scale ranging from 0 (not true) to 4 (almost always true). The items are divided into three subscales of work resilience: individual, direct supervisor, and senior management. Each subscale’s score was calculated as the average of its items. The questionnaire is valid and provides a mean workplace resilience score to illustrate differences between groups [32]. The Cronbach’s alpha value of the Resilience at Work index was 0.86.

#### Threat perception questionnaire (Kimhi & Eshel, 2012)

This study used the Kimhi & Eshel questionnaire to assess individual threat perception. The item wording in the questionnaire was modified to fit the specific threats being studied. The respondents were asked to rank six threats that the hospital must prepare for: MCIs, MTIs, UBEs, cyber-attacks, earthquakes, and war. Responses are given on a 5-point Likert scale ranging from 1 (not threatening at all) to 5 (very highly threatening) [33]. The threat perception score was calculated as the average of the six items.

### Statistical analysis

Descriptive statistics were used to present the frequencies and percentages of categorical variables. Continuous variables were described using measures of central tendency (median, mean) and dispersion (range, variance, standard deviation) was used to describe the demographic and occupational characteristics of the participants. In the bivariate analysis, Pearson’s correlation coefficient was calculated to measure the strength and direction of the linear relationship between two continuous dependent and independent variables. A series of T-tests were used to examine mean differences between two independent samples, and ANOVA tests were conducted to assess mean differences across multiple categorical groups. Relationships between categorical variables were examined using the Chi-square test. Furthermore, a linear regression analysis was conducted to assess predictors of well-being, individual resilience, and resilience at work (two linear regressions for each). Each variable was predicted using the other two dependent variables. Additionally, each variable was predicted using the following variables as appropriate: age, gender (females compared to males), well-being, individual resilience, resilience at work, average threat perception, born in Israel (compared to others), religion (Jews compared to others), marital status, the interaction between gender (female) and marital status (in a relationship), levels of religiosity (secular compared to traditional, religious and very highly religious), education (academic compared to non-academic), profession (nurses compared to physicians and administration including auxiliary staff), experience (11 years and above compared to 0–10 years), type of department (emergency department and internal department compared to others), managerial position, weekly working hours, training in the last five years, and reserve duty since the Hamas attack on October 7, 2023. Each variable was first predicted using all variables (Model 1) and then using the optimal number of essential predictive variables, regulated to age and gender (Model 2). This two-model approach enabled a comparison between a detailed, full analysis (Model 1) and a more streamlined model (Model 2), providing clearer insights into the key predictors for each outcome. AMOS software was utilized to generate a visual representation, providing a more abstract illustration of the model for clearer conceptual understanding. All statistical procedures were conducted using SPSS version 28, with a significance threshold of *p* < 0.05.

## Results

A total of 434 hospital employees completed the questionnaire. The age distribution of the participants was fairly even between younger and older employees. Among the participants, 108 (25.3%) were men and 319 (74.7%) were women. Most participants were born in Israel, identified as Jewish, and were in a relationship, with a large majority having children and an academic education. Additionally, 218 (~ 52%) respondents identified themselves as traditional, religious, or very highly religious. Approximately 82% of the respondents were health professionals. The experience levels among participants were almost evenly divided between those with less than ten years and those with more than ten years of experience. Most participants worked over 40 h per week, and the majority were regular employees, with a smaller proportion holding managerial positions. About 13.8% of the respondents worked in internal medicine departments, 11.1% in emergency departments, and 75.1% in other departments. Furthermore, ~ 67% of the respondents had received at least one emergency training. The participants’ characteristics are presented in Table 1.

**Table 1:** Demographic and professional characteristics (n = 434)

Category		Frequency	Valid percent	Mean	Std. deviation
Age	≤ 42	216	50.8	42.53	11.65
	> 42	208	49.0		
Gender	Male	108	25.3	.75	.43
	Female	319	74.7		
Country of birth	Born in Israel	292	69.0	.69	.46
	Other	131	31.0		
Religion	Jewish	368	87.8	.87	.32
	Other	51	12.2		
In a relationship	No	101	23.7	.76	.42
	Yes	326	76.3		
Children	No	68	15.9	.84	.36
	Yes	359	84.1		
Religiosity	Secular	203	48.2	1.68	.74
	Traditional	146	34.7		
	Religious and very highly religious	72	17.1		
Academic education	No	61	14.5	.85	.35
	Yes	360	85.5		
Sector	Physicians	102	25.3	1.92	.65
	Nurses	228	56.6		
	Administration including auxiliary staff	73	18.1		
Experience	0–10 year	193	46.8	.53	.49
	11 + years	219	53.2		
Weekly working hours	≤ 40	112	27.3	41.52	11.26
	> 40	292	71.8		
Department	Emergency	48	11.1	2.64	.67
	Internal	60	13.8		
	Others	326	75.1		
Position	Regular employee	312	75.7	.24	.429
	Managerial	100	24.3		
Emergency training	No	136	33.0	.66	.47
	Yes	276	67.0		
Reserve duty since October 7	No	390	94.7	.05	.22
	Yes	22	5.3		

### Levels of threat perceptions

**Fig. 1:**
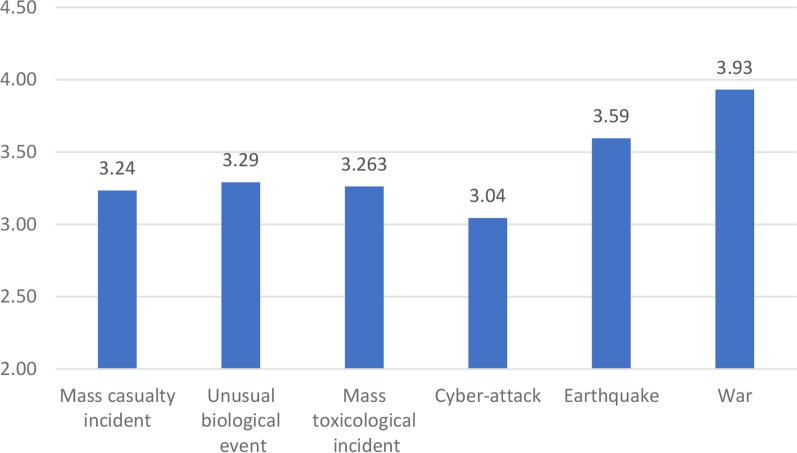
The mean perceived threats

Wars were reported as the highest perceived threat, whereas cyber-attacks were considered the least threatening. The mean perceived threats are presented in Fig. 1.

### Levels of well-being, individual and workplace resilience

The mean score for well-being was M ± SD = 10.43 ± 4.9. Individual resilience had a mean score of M ± SD = 5.97 ± 1.5, while resilience at work exhibited a mean score of M ± SD = 27 ± 7.1.

### Associations between perceived threats, individual resilience, resilience at work, and well-being

Pearson’s correlation coefficients were calculated to assess the relationships between different variables. The association between well-being and individual resilience was r = 0.3 (*p* < 0.001), between well-being and resilience at work was r = 0.26 (*p* < 0.001), and between individual resilience and resilience at work was r = 0.33 (*p* < 0.001). Several types of threats were found to be negatively associated with both well-being and individual resilience, including wars, MCIs, and cyber-attacks. MTIs, unusual biological events (UBEs), and earthquakes were significantly negatively associated only with well-being. Resilience at work, however, was not found to correlate with any of the perceived threats. See Table 2.

**Table 2:** Associations between Perceived Threats, Resilience at Work, Individual Resilience and Well-being

	Well-bring	Individual resilience	Resilience at work
Mass casualty incident	− 0.16***	− 0.14**	− 0.04
Mass toxicological incident	− 0.13**	− 0.03	0.03
Unusual biological event	− 0.11*	− 0.006	0.05
Cyber-attrack	− 0.18***	− 0.14**	− 0.04
Earthquake	− 0.2***	− 0.08	0.005
War	− 0.25***	− 0.17***	− 0.05
Well-bring	1	0.3**	0.26***
Individual resilience	0.3***	1	0.33***
Resilience at work	0.26***	0.33***	1

*p < 0.05; **p < 0.01; ***p < 0.001

### Variance in well-being, individual and work resilience

The data revealed significant differences in well-being according to demographic or employment characteristics. Differences were observed according to gender, with men reporting significantly higher mean levels of well-being than women (M ± SD = 12.25 ± 5.4 versus M ± SD = 9.82 ± 4.7 respectively T-test; *p* < 0.001). Physicians compared to nurses and administration teams presented significantly higher mean levels of well-being (M = 12.25, 9.65, 10.27 respectively ANOVA; *p* < 0.001). Participants not in a relationship reported higher well-being than those in a relationship (M ± SD = 11.4 ± 5.6, M ± SD = 10.1 ± 4.7 respectively T-test; *p* = 0.039). Secular, traditional, and religious or very highly religious individuals also showed significant differences in well-being (M = 10.42, 9.9, 11.86 respectively ANOVA; *p* = 0.02). Additionally, those with 0–10 years of experience reported higher well-being than those with 11 + years (M ± SD = 11.3 ± 5.2, M ± SD = 9.9 ± 4.7 respectively T-test; *p* = 0.003), and individuals not on reserve duty since October 7 exhibited higher well-being compared to those who were on reserve duty (M ± SD = 10.4 ± 4.9, M ± SD = 13 ± 5.8 respectively T-test; *p* = 0.017).

Individuals’ resilience analysis indicated that respondents born outside Israel had a higher mean level of individual resilience compared to others (M ± SD = 6.13 ± 1.4, M ± SD = 5.7 ± 1.7 respectively T-test; *p* = 0.006). Concerning work resilience differences were observed according to Participants not in a relationship than those in a relationship (M ± SD = 24.6 ± 8.4, M ± SD = 27.7 ± 6.6 respectively T-test; *p* < 0.001). Secular, traditional, and religious or very highly religious individuals also showed significant differences in work resilience (M = 25.3, 28.3, 28.6 respectively ANOVA; *p* < 0.001). Additionally, physicians compared to nurses and administration teams presented significantly higher mean levels of work resilience (M = 26, 26.4, 29.4 respectively ANOVA; *p* = 0.003). Variables such as having children, level of education, employment position, and the department in which the respondents worked did not significantly contribute to the explained variance in well-being, individual resilience, or resilience at work. The descriptive statistics and analysis are presented in Table 3.

**Table 3:** Descriptive statistics and analysis of well-being, individual resilience, and workplace resilience across demographic and professional characteristics

			Well-being		Individual resilience		Resilience at Work	
Variables		N	M ± SD	*P* value	M ± SD	*P* value	M ± SD	*P* value
Gender	Male	108	5.4 ± 12.25	T (425) = 4.48 , *p* < 0.001***	1.6 ± 5.99	T (425) = 0.11 , *p* = 0.91	7.62 ± 25.85	T (425) = 1.85 , *p* = 0.066
	Female	319	4.7 ± 9.82		1.4 ± 5.97		6.9 ± 27.32	
Country of birth	Born in Israel	292	5.1 ± 10.58	T (421) = 0.88 , *p* = 0.38	1.4 ± 6.13	T (421) = 2.77 , *p* = 0.006**	7.4 ± 26.93	T (421) = 0.13 , *p* = 0.89
	Other	131	4.9 ± 10.12		1.7 ± 5.66		6.8 ± 27.04	
In a relationship	No	101	5.6 ± 11.41	T (425) = 2.08 , *p* = 0.039*	1.7 ± 5.8	T (425) = 1.33 , *p* = 0.18	8.4 ± 24.58	T (425) = 3.41 , *p* < 0.001***
	Yes	326	4.7 ± 10.13		1.4 ± 6.03		6.6 ± 27.68	
Religiosity	Secular	203	10.42	F (2, 418) = 3.94 , *p* = 0.02*	5.99	F (2, 418) = 0.38 , *p* = 0.68	25.3	F (2, 418) = 10.67 , *p* < 0.001***
	Traditional	146	9.86		5.92		28.3	
	Religious and very highly religious	72	11.86		6.11		28.6	
Sector	Physicians	102	12.25	F (2, 400) = 10.2 , *p* < 0.001***	6.14	F (2, 400) = 1.86 *p* = 0.16	26	F (2, 400) = 5.86, *p* = 0.003**
	Nurses	228	9.65		5.9		26.4	
	Administration including auxiliary staff	73	10.3		6.16		29.4	
Experience	0–10 year	193	5.2 ± 11.25	T (410) = 2.95 , *p* = 0.003**	1.5 ± 6.02	T (410) = 0.29 , *p* = 0.77	7.8 ± 26.3	T (410) = 1.45 , *p* = 0.15
	11 + year	219	4.2 ± 9.82		1.4 ± 5.98		6.7 ± 27.3	
Reserve duty since October 7	No	390	4.9 ± 10.35	T (410) = 2.4 , *p* = 0.017*	1.5 ± 5.98	T (410) = 1.04 , *p* = 0.29	7.1 ± 26.97	T (410) = 1.33 , *p* = 0.18
	Yes	22	5.8 ± 12.95		1.4 ± 6.32		8.5 ± 24.89	

*p < 0.05; **p < 0.01; ***p < 0.001

#### Factors associated with well-being, individual and work resilience

**Fig. 2:**
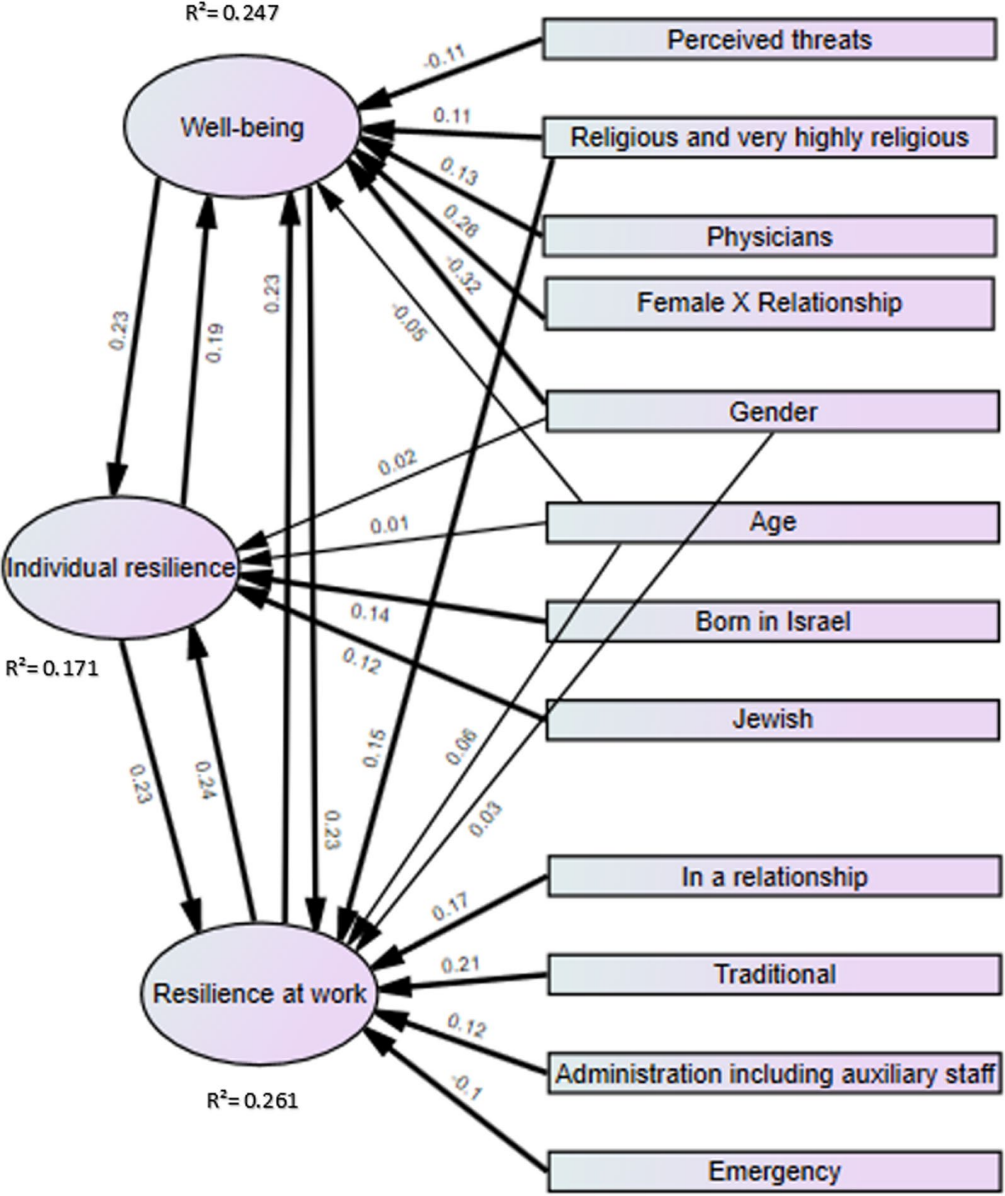
Path analysis regression Model 2. *Note*: Thick arrows define statistically significant results

In analyzing the factors associated with well-being, individual resilience, and resilience at work, we compared two regression models: Model 1 (all variables) and Model 2 (optimal variables). The interaction between gender (female) and marital status (in a relationship) was tested across all variables. In the model predicting well-being, the interaction was significant and included in both predictor models. However, for personal resilience and work resilience, the interaction was not significant in either model and was consequently excluded from those models. The regression analysis’s results are presented in Table 4 and Fig. 2.

**Table 4:** Regression analysis of model 1

Well-being			Individual resilience			Resilience at work		
Model 1			Model 1			Model 1		
Variable	β	*p*. v	Variable	β	*p*. v	Variable	β	*p*. v
Resilience at work	0.23	< 0.001**	Well-being	0.258	< 0.001**	Traditional	0.208	< 0.001***
Individual resilience	0.21	< 0.001**	Resilience at work	0.221	< 0.001**	Well-being	0.231	< 0.001***
Female gender	− 0.34	< 0.001**	Born in Israel	0.155	0.002**	Individual resilience	0.234	< 0.001***
Marital status	− 0.24	0.004*	Jewish	0.084	0.13	Marital status	0.179	0.001***
Physicians	0.17	0.005*	Average threat perception	− 0.084	0.097	Religious and very highly religious	0.15	0.004**
Female X relationship	0.28	0.014*	Female gender	0.072	0.227	Emergency department	− 0.091	0.059
Average threat perception	− 0.10	0.034*	Age	0.037	0.632	Administration including auxiliary staff	0.114	0.064
Religious and very highly religious	− 0.11	0.04	Marital status	0.028	0.633	Emergency training	0.072	0.13
Born in Israel	− 0.07	0.16	Traditional	− 0.039	0.472	Age	0.086	0.24
Emergency department	− 0.07	0.17	Religious and very highly religious	− 0.063	0.256	Jewish	− 0.054	0.307
Managerial role	0.07	0.21	Academic education	− 0.022	0.726	Reserve duty since October 7, 2023	− 0.042	0.395
Weekly working hours	− 0.06	0.22	Physicians	− 0.021	0.74	Female gender	0.045	0.428
Jewish	− 0.07	0.23	Administration including auxiliary staff	− 0.046	0.47	Academic education	− 0.042	0.482
Administration including auxiliary staff	0.07	0.28	Seniority of 11 years and above	− 0.002	0.98	Physicians	0.024	0.689
Internal department	− 0.04	0.37	Emergency department	0.09	0.076	Weekly working hours	0.019	0.71
Traditional	− 0.04	0.43	Internal department	− 0.03	0.547	Seniority of 11 years and above	− 0.025	0.722
Age	− 0.05	0.49	Managerial role	− 0.062	0.275	Internal department	− 0.014	0.771
Academic education	0.04	0.53	Weekly working hours	− 0.013	0.807	Managerial role	− 0.011	0.84
Reserve duty since October 7, 2023	0.03	0.56	Emergency training	− 0.03	0.553	Born in Israel	− 0.006	0.896
Emergency training	− 0.02	0.68	Reserve duty since October 7, 2023	0.02	0.67	Average threat perception	− 0.001	0.977
Seniority of 11 years and above	− 0.03	0.69	F (20, 361) = 6.42, *p* < 0.001 **R**^**2**^** = 0.197**, adj = 0.152			F (20, 361) = 6.79, *p* < 0.001 **R**^**2**^** = 0.273**, adj = 0.233		
F (21, 360) = 6.22, *p* < 0.001 **R**^**2**^** = 0.266**, adj = 0.223								

*p < 0.05; **p < 0.01; ***p < 0.001

#### Factors associated with well-being

Concerning well-being, the findings indicate that In Model 1, 23.8% of the variance was attributed to resilience at work (*p* = 0.001) and individual resilience accounted for 20.6% of the variance (*p* = 0.001). These results were consistent in Model 2, demonstrating that higher levels of individual resilience and resilience at work were associated with improved well-being. Physicians had a positive beta value (0.159) in Model 1 and remained significant in Model 2, suggesting that being a physician is associated with higher well-being. Conversely, higher threat perception was linked to reduced well-being. Gender was a notable factor with female gender showing a negative beta value (− 0.167) in both models. However, when the interaction between gender (female) and relationship was examined, a significant positive relationship with well-being was found in both Model 1 (β = 0.209) and Model 2 (β = 0.196). In addition, religious and very religious individuals exhibited a positive beta value (0.11) in both models, indicating that religiosity is associated with higher well-being. Although not statistically significant, age showed a slight negative trend in the regression, suggesting that older participants may experience a marginal decrease in well-being. Academic education did not have a significant effect on well-being. Model 1 incorporates 20 predictors and accounts for 25.4% (R^2^ = 0.254), of the variance in well-being. In contrast, Model 2 simplifies the analysis by focusing on the 7 most significant predictors, explaining 23.1% (R^2^ = 0.231) of the variance in well-being.

#### Factors associated with individual resilience

The findings reveal that individuals who report higher levels of resilience at work and better well-being tend to exhibit greater individual resilience. Approximately 22% of the variance in individual resilience is explained by resilience at work, and about 26% is accounted for by well-being. These results were consistent also in Model 2, demonstrating that higher levels of well-being and resilience at work were associated with improved individual resilience. Additionally, being born in Israel significantly predicted individual resilience in both models. Identifying as Jewish was a significant predictor in Model 2, suggesting a positive association with individual resilience. Although not statistically significant, average threat perception showed a negative trajectory, implying that higher threat perception might be associated with lower individual resilience. Other variables, such as gender, age, and marital status, did not significantly influence individual resilience in either model. Model 1 explains 19.7% (R^2^ = 0.197), of the variance with 20 predictors. Model 2, however, uses only 6 significant predictors and explains 17.1% (R^2^ = 0.171), of the variance.

#### Factors associated with resilience at work

Individuals who reported higher levels of well-being and individual resilience also exhibited greater resilience at work. Well-being and individual resilience accounted for approximately 23% of the variance in resilience at work. Furthermore, individuals in relationships, as well as those who are traditional and highly religious, demonstrated higher levels of resilience at work. Employment in administrative roles, including auxiliary staff, was associated with increased resilience, while working in the emergency department showed a significant negative relationship with resilience at work, resulting in a 10% decrease. Although not statistically significant, participation in emergency training indicated a positive trajectory, suggesting it may be associated with enhanced resilience at work. In examining resilience at work, Model 1 includes 20 predictors, explaining 27.3% (R^2^ = 0.273), of the variance. Model 2 narrows the scope to the 9 most significant predictors, accounting for 26.1% (R^2^ = 0.261), of the variance. Across all three constructs, Model 2 consistently demonstrated superior performance and utility compared to Model 1. Despite a slight reduction in explained variance, Model 2 offers a more parsimonious and interpretable model, highlighting key factors with greater statistical robustness. The reduction in the number of predictors in Model 2 leads to a more focused model, enhancing clarity and mitigating potential multicollinearity issues.

## Discussion

The aim of this study was to examine how emergency responders’ perception of threat relates to well-being, individual resilience, and resilience at work among staff in a general hospital. The main findings indicate that higher levels of resilience at work and well-being are associated with greater individual resilience. Being born in Israel significantly predicted individual resilience, as did identifying as Jewish. Higher threat perception was negatively correlated with both well-being and individual resilience. Gender emerged as a significant predictor of well-being, with men reporting higher well-being than women. Marital status was a significant predictor of women’s well-being, with women in relationships reporting higher levels of well-being compared to those not in relationships. The professional sector was also relevant, with physicians reporting higher well-being and administrative staff demonstrating higher resilience at work compared to nurses. Conversely, employment in the emergency department showed a significant negative relationship with resilience at work**.**

The study’s findings highlight several substantial factors that influence well-being, individual resilience, and resilience at work in the context of Israeli healthcare personnel. First, the fact that war was perceived as the highest threat among the investigated emergency scenarios and had the most significant negative impact on the well-being of hospital staff can most likely be attributed to the unexpected Hamas assault on Israel on October 7, 2023 [34]. The attack significantly altered the psychological and emotional landscape of the nation and created an intense and stressful environment affecting the entire Israeli population.[35] The ongoing conflict and fear of the war expanding to other fronts have probably contributed to a significant increase in the perception of the war threat, contributing to the decline in the well-being of the population [36]. Studies conducted during similar periods of conflict have shown that the constant threat of war and the accompanying uncertainty severely impact the well-being of both civilians and professionals [37].

The lack of difference in the well-being of emergency department teams (directly involved in treating war casualties) versus internal medicine staff (less exposed to war injuries) may be attributed to the timing of the study as well. The shared exposure to trauma and the broad nature of the crisis created a common burden of stress and trauma [38]. This effect has been observed in other high-stress situations where typical departmental distinctions blur due to the collective crisis [39]. The study found lower well-being levels among women, consistent with previous studies that have consistently shown that women tend to exhibit lower well-being levels following traumatic events, experiencing higher levels of anxiety, depression, and stress [40]. However, women in relationships exhibited higher levels of well-being compared to those not in a relationship. Several studies have specifically examined the influence of marital or romantic relationships on women’s well-being, consistently demonstrating that women derive considerable emotional support and social benefits from these partnerships, which in turn significantly enhance their overall well-being [41]. Regarding life satisfaction levels, the study found that physicians reported higher levels of well-being compared to nurses. This difference can be attributed to several key factors, such as salary and education. Physicians generally earn higher salaries, which can contribute to overall life satisfaction. Financial stability and the ability to afford a higher standard of living can alleviate many stressors and improve well-being [42, 43].

In line with previous findings, this study found a positive relationship between individual resilience, resilience at work, and well-being [44]. People with higher well-being are better equipped to face challenges, bounce back from setbacks, and maintain a positive outlook in difficult situations [45]. In addition, religiosity showed a positive relationship with well-being and resilience at work, especially during times of crisis. This relationship is supported by previous research emphasizing the role of spiritual beliefs and practices in providing emotional support and a sense of purpose. These elements are essential in times of high stress and uncertainty, such as those experienced during conflicts or natural disasters. Faith helps people to feel connected to ‘something bigger’, which can be comforting and stabilizing [46]. The positive relationship between resilience at work and individual resilience supports the idea that resilience in one aspect can strengthen resilience in other aspects, creating a synergistic effect [47]. The highest individual resilience was observed among Israeli-born, which can be attributed to Israel’s unique sociopolitical context and frequent exposure to conflicts and hardships. Israel has a history characterized by wars, terror events, and ongoing security threats, from the struggle to establish the state of Israel to date. This historical narrative fosters a collective culture of resilience, cohesion, and strong adaptability among Israelis [48, 49]. Based on the salutogenic theory, the sense of coherence enables Israelis to often perceive their environment as comprehensible and manageable despite its challenges [50, 51]. Furthermore, the "Rally around the flag" effect during times of national crisis, such as October 7, enhances the sense of coherence, fostering national solidarity and a collective identity that strengthens personal and societal resilience [52].

Resilience at work was also positively predicted by factors such as marital status, sector, and type of department. The support and stability provided by a relationship can enhance an individual’s ability to cope with workplace stressors and maintain a positive work point of view [53]. The study’s results also indicate that administration, including auxiliary staff, had higher resilience at work compared to nurses. This sector, unlike nursing, is less exposed to high-intensity stressors, allowing them to maintain higher resilience at work. Reduced exposure to high-stress environments may enable these workers to maintain better well-being and resilience [54]. The emergency department was found to have a negative effect on resilience at work, which can be attributed to the nature of the department, which is characterized by high-stress, high-intensity situations that can lead to burnout and reduced resilience [55]. The continuous need to make quick life-saving decisions can be mentally and physically exhausting. High-intensity work environments are associated with higher burnout rates and lower resilience among healthcare workers [56]. Additionally, they are often exposed to traumatic events, which can undermine resilience over time. This exposure can lead to secondary traumatic stress and reduce their ability to cope effectively, highlighting the importance of maintaining resilience among healthcare workers in these departments [57].

The average perception of threat was not found to be a predictor of personal and work resilience. This may be because the perception of threat is already integrated into the broader perception of well-being, which has a significant positive relationship with resilience measures. Previous studies have indicated that the psychological stress and exposure to traumatic events typically experienced by emergency service workers do not necessarily predict their resilience level but are closely linked to their overall mental health status [58]. Therefore, while the perception of threat alone may not serve as an independent predictor, it is an integral part of the complex interplay of factors contributing to well-being and resilience [59].

## Limitations

While this study provides important insights into the relationship between threat perceptions, well-being, individual resilience, and resilience at work, it is essential to consider the inherent limitations in its design and execution. First, the study was conducted one month after the unexpected Hamas assault, and it should be taken into consideration that this event substantially impacted the respondents. Also, during the data collection period, many staff were called to reserve duty due to the ongoing war, which led to a decrease in their availability in the wards. This reduced availability may have affected the response rate and could have increased the stress levels in the departments. Although people who served in reserve duty reported lower levels of well-being and higher exposure to traumatic events, this group was small, and thus, it was not possible to achieve significant results when comparing their response to that of the other respondents. As a result, no definitive conclusions could be drawn from their responses. Future measurements after the war is resolved may yield different and potentially more significant results. Additionally, there may be bias between responders and non-responders to the survey, as those who participated might exhibit different levels of resilience or threat perception compared to those who did not. Furthermore, the CD-RISC-2 questionnaire for measuring individual resilience, which includes only two items, may be too limited to capture the full variance of the construct being measured. Furthermore, the reliance on self-report measures introduces potential biases. Participants’ responses may have been influenced by social desirability bias, leading them to present themselves in a more positive light. Finally, the generalizability of the findings is limited. Israel’s frequent exposure to conflict has fostered a unique culture of resilience, which may not be as prevalent in other countries. The findings are specific to the context and reflect Israel’s unique social, political, and cultural environment in a time of unexpected conflict. As such, it is possible that the results may not be applicable to other societies or contexts that experience different stressors. Future research in other settings, particularly in countries not regularly exposed to conflict, would be necessary to determine whether similar patterns of resilience and well-being can be observed elsewhere.

## Conclusions

The research conducted shortly after Hamas’s attack on Israel during the Israel-Gaza war offers important insights on the factors influencing well-being, individual resilience, and workplace resilience among hospital staff in conflict situations. The immediate threat of war was perceived as the most significant, illustrating how threat perceptions and their impacts evolve based on the context and timing of the study. Prolonged emergencies can severely affect well-being, highlighting the necessity for timely psychological support and interventions. Israel’s frequent exposure to conflicts cultivates a culture of resilience and adaptability. While short-term individual resilience can be bolstered by a sense of coherence and collective solidarity, these effects may vary in the long-term depending on the crisis’s nature and intensity.

The study identified key predictors of well-being, individual resilience, and workplace resilience, emphasizing the complexity of these relationships and the need for a comprehensive approach. Threat perception directly impacts well-being, indirectly affecting personal and workplace resilience through its influence on well-being. The interconnection between personal and work resilience and well-being underscores the importance of integrated programs that enhance individual well-being and foster resilience in both personal and professional domains. The positive role of religiosity in improving well-being and resilience at work suggests that finding purpose and meaning during crises can enhance emotional stability. The study also highlighted significant gender differences, with women experiencing lower well-being after traumatic events, necessitating targeted interventions for women, especially in high-stress professions like healthcare. Additionally, the disparity in life satisfaction between physicians and nurses, influenced by factors such as salary and education, indicates a need for systemic organizational changes. The varying resilience levels among medical staff call for sector-specific interventions to address the unique challenges faced by different job positions. Therefore, specialized training programs are essential to equip teams with the skills needed to manage stress and maintain resilience during emergencies. Future research should examine the impact of emergency training on resilience. Based on these findings, the importance of cultivating well-being and resilience, both personally and professionally, becomes vital in creating a strong and resilient medical system that can effectively manage future crises.

### Implications of findings for the determination and formulation of health policy

The unpredictable and extended nature of contemporary conflicts calls for a reassessment of health policy and resilience strategies. This study’s findings indicate that conventional approaches to healthcare resilience may be inadequate in addressing the significant challenges encountered by hospital staff in conflict zones. Rather than depending on reactive measures that target well-being and resilience, policies should prioritize embedding resilience-building mechanisms into the core operations of healthcare organizations. In Israel, where resilience is deeply ingrained due to frequent conflicts, there is a distinct opportunity to institutionalize resilience programs within hospital systems. This can be accomplished by creating sustainable resilience units within hospitals. These units would serve as dedicated support centers, providing ongoing access to psychological counseling, stress management, and peer support. Unlike current resilience and stress management initiatives, these units would embed resilience efforts into routine hospital operations, while simultaneously evaluating and adapting these programs. This approach would establish a feedback loop, enabling hospitals to make real-time adjustments based on staff well-being data. It is recommended that hospitals establish personal development groups where staff can set both personal and professional goals within a supportive and collaborative environment. Facilitated by mentors or coaches, these groups would enable healthcare workers to concentrate on their aspirations, whether they pertain to career advancement or personal well-being. To address the significant gender differences identified in the study, health policy should prioritize targeted interventions for women. Mental health services in hospitals are frequently generalized and overlooking gender-specific stressors faced by women. Therefore, it is crucial to develop dedicated mental health services tailored to female healthcare workers, including trauma-informed therapy, gender-sensitive stress management workshops, and peer support networks specifically for women. These targeted interventions should also encompass leadership development programs and flexible scheduling options that accommodate personal and family responsibilities, ensuring that female staff receive the support necessary to thrive. In the field of nursing, it is recommended to establish a comprehensive policy addressing remuneration and professional development. Though the study was conducted in one specific Israeli hospital, the same health policy recommendations may be applicable to global healthcare entities at large and particularly hospitals in areas afflicted by conflicts. As the challenges that were highlighted are most probably shared by hospitals who operate in risk areas, the recommended solutions, such as the need for personal development support groups, gender-specific mental health programs, and leadership development interventions, will contribute to the resilience and fortitude of these medical entities beyond the hospital that was surveyed.

The implementation of these targeted, comprehensive resilience strategies will not only fortify healthcare systems in conflict zones but also foster a more sustainable and supportive environment for healthcare professionals.

## Data Availability

Due to ethical considerations, the raw data are not uploaded in a public repository. The data that support the findings of this study are available from the corresponding author upon reasonable request.

## Abbreviations

CD-RISC 2: Connor-Davidson resilience scale
COVID-19: Corona Virus Disease 2019
CRED: Center for Research on the Epidemiology of Disasters
MCI: Mass casualty incident
MHC-SF: Mental Health Continuum-Short Form
SARS: Severe acute respiratory syndrome
MTI: Mass toxicological casualty incident
UBE: Unusual biological event
